# Eradication of *Klebsiella pneumoniae* pulmonary infection by silver oxytetracycline nano-structure

**DOI:** 10.1186/s13568-024-01720-5

**Published:** 2024-05-29

**Authors:** Farag M. Mosallam, Rana Elshimy

**Affiliations:** 1 Drug Radiation Research Department, Microbiology Lab, Biotechnology Division, National Center for Radiation Research and Technology (NCRRT), Egyptian Atomic Energy Authority, Cairo, Egypt; 2 Microbiology and Immunology, Faculty of Pharmacy, AL-Aharm Canadian University (ACU), Giza, Egypt; 3 Microbiology and Immunology, Egyptian Drug Authority, Cairo, Egypt

**Keywords:** *Klebsiella pneumoniae*, Antibiotic resistance, Ag-OTC-Ns, In vivo antimicrobial efficacy

## Abstract

Targeted bactericidal nanosystems hold significant promise to improve the efficacy of existing antimicrobials for treatment of severe bacterial infections by minimizing the side effects and lowering the risk of antibiotic resistance development. In this work, Silver Oxytetracycline Nano-structure (Ag-OTC-Ns) was developed for selective and effective eradication of *Klebsiella pneumoniae* pulmonary infection. Ag-OTC-Ns were prepared by simple homogenization-ultrasonication method and were characterized by DLS, Zeta potential, TEM and FT-IR. The antimicrobial activity of Ag-OTC-Ns was evaluated in vitro using broth micro-dilution technique and time-kill methods. Our study showed that MICs of AgNO_3_, OTC, AgNPs and Ag-OTC-Ns were 100, 100, 50 and 6.25 µg/ml, respectively. Ag-OTC-Ns demonstrated higher bactericidal efficacy against the targeted *Klebsiella pneumoniae* at 12.5 µg/ml compared to the free Oxytetracycline, AgNO_3_ and AgNPs. In vivo results confirmed that, Ag-OTC-Ns could significantly eradicate *K. pneumoniae* from mice lung in compare with free Oxytetracycline, AgNO_3_ and AgNPs. In addition, Ag-OTC-Ns could effectually diminish the inflammatory biomarkers levels of Interferon Gamma and IL-12, and as a result it could effectively lower lung damage *in K. pneumoniae* infected mice. Ag-OTC-Ns has no significant toxicity on tested mice along the experimental period, there was no sign of behavioral abnormality in the surviving mice indicating that the Ag-OTC-Ns is safe at the used concentration. Furthermore, capability of 5 kGy Gamma ray to sterilize Ag-OTC-Ns solution without affecting it stability was proven.

**Supplementary Information:**

The online version contains supplementary material available at 10.1186/s13568-024-01720-5.

## Introduction

*Klebsiella pneumoniae* is a gram negative bacterium that’s commonly related with respiratory infections, particularly in clinic settings (McDaniel and Allen 2019). *K. pneumoniae* has a great resistance rate to most of the commonly utilized antibiotics and is reported as one of ESKAPE pathogens that are in an urgent need for a novel efficient antimicrobial (Effah et al. 2020). Oxytetracycline is a broad tetracycline anti-microbial agent that acts on a huge number of gram positive and gram negative microbes by interfering with bacterial protein synthesis but unfortunately Oxytetracycline is incompletely and irregularly absorbed from the gastrointestinal tract (Mog et al. 2020). Oxytetracycline is utilized to treat infections of the respiratory tract (pneumonia), urinary tract, delicate tissues, and skin (Pickens and Tang 2010).

Antimicrobial resistance has been identified as one of the three major problems countered in human health by the World Health Organization (WHO) (Siddique et al. 2020). Particularly nanoparticles, which are nanostructured biomaterials, possess special physicochemical characteristics including significant surface area, high reactivity, functionalizable structure, and ultra-small and controlled size (Shimanovich and Gedanken 2016). The utilization of nanostructures with ordinary antibiotics has been demonstrated to enhance their antimicrobial activity against drug resistant microbial strains (Jampilek and Kralova 2022).

The antibacterial effects of medicines can be enhanced by combining NPs with other structures, or by packaging many medications or antimicrobials into a single NP. The combinatorial approach can be used to extend the effective time, which can effectively and significantly reduce the probability that bacteria would develop resistance (Brooks and Brooks 2014). The size, charge, zeta potential, surface shape, and crystal structure of NPs are among its physicochemical characteristics, and these factors play a crucial role in controlling how NPs interact with bacterial cells. In addition, the bacterial strain, exposure duration, and environmental factors are other important elements that affect how effective NPs are against bacteria (Çalışkan et al. 2014).

Therefore, the present study was designed for preparation of Ag-OTC-Ns to improve Oxytetracycline, AgNPs activity, decrease their side effects and eradicate *Klebsiella pneumonia* pulmonary infection*.* The in vitro and in vivo antibacterial efficacy of the Ag-OTC-Ns against clinical isolates of multidrug resistant *Klebsiella pneumonia* was investigated. The biostatic and bactericidal effects of the Ag-OTC-Ns against *K. pneumoniae* were conducted to determine in vitro and in vivo efficacy.

## Material and methods

### Materials

Chemicals (Oxytetracycline from CID company, EGYPT and; others chemicals and reagents utilized in the following examinations and biological experiments were received at analytical standard grade (Sigma-Aldrich), and appropriated externally for additional purification.

### Methods

#### Preparation and validation of silver oxytetracycline nano-structure

Silver Oxytetracycline Nano-structure (Ag-OTC-Ns), were prepared by simple homogenization-ultrasonication method as previous described (Fernandes et al. 2016; Mosallam et al. 2023) with some modification. Briefly, a suspension of silver nitrate (100 µg/ml) was prepared in deionized water and added drop wise to mixture of Oxytetracycline (100 µg/ml prepared in DMSO) and 10% surfactant. The final mixture were mixed by homogenization (at 15,000 rpm for 30 min) and then sonicated for 15 min using ultrasonic processor using a cooling bath to maintain the low temperature.

For characterization of Ag-OTC-Ns, several validations were used including particle size and particle size distribution. The stability of Ag-OTC-Ns was determined by Zeta potential using Zetasizer Technique (PSS-NICOMP 380-ZLS, USA) and particles size distribution using DLS. In addition, Transmission Electron Microscopy (TEM) was used to measure the particle size of Ag-OTC-Ns, while the Fourier transform infrared spectroscopy (FT-IR) was employed to assess the function moiety (El-Batal et al. 2020).

Radiation treatments: Silver Oxytetracycline Nano-structure samples were sealed in plastic tubes and exposed to gamma rays at dose levels of 0, 5, 10 and 20 kGy at NCRRT, Cairo, Egypt; to study the impact of gamma radiation on Silver Oxytetracycline Nano-structure stability and sterility.

### In vitro antibacterial assay

#### Microorganism and culture media

*Klebsiella pneumoniae* strains: In this study *K. pneumoniae* ATCC 13883 (stander strain), in addition to clinical MDR isolates of *K. pneumonia* were used. The standard strain was kindly gifted by Regional Center for Mycology and Biotechnology (RCMB), Al-Azhar University, Cairo, Egypt, while clinical isolates were refreshed from the stock culture collection of Microbiology Lab., Drug Radiation Research Department, Biotechnology Division, NCRRT, EAEA, Cairo, Egypt. The isolate strain were selected based on higher MDR among 10 clinical isolates screened especially more resistant against free oxytetracycline. For the agar diffusion experiments, sabouraud Dextrose Agar (Oxoid) and or Brain Heart Infusion Agar (BHI agar) were employed, while the Muller-Hinton broth (CAMHB, Oxoid, Basingstoke, UK) was used for the minimal inhibition concentration (MIC). Mackonkey agar was used for lung count *K. pneumoniae*.

#### Agar well-diffusion method

Preliminary screening of antibacterial activities of Silver Oxytetracycline Nano-structure was first screened for inhibitory zone diameter (IZD) by the agar well diffusion method. Agar well diffusion method is widely used to evaluate the antimicrobial activity of antimicrobial agents (Magaldi et al. 2004). The Brain Heart Infusion Agar (BHI agar) plates’ surfaces were inoculated by spreading a volume of the microbial inoculum over the entire agar surface. Then, a hole with a diameter of 6 mm was punched aseptically with a sterile corkborer and a volume 50 µl of the AgNO_3_, OTC and Ag-OTC-Ns at desired concentration (100 µg/ml) was introduced into the well. After that, agar plates were incubated under at 37 °C for 24 h. The antimicrobial agent diffused in the agar medium and the inhibitory zones were measured in mm.

### Determination of MIC

The minimal inhibitory concentration (MIC) is the lowest concentration of antimicrobial agent that completely inhibits growth of the organism in tubes or micro-dilution wells as detected by the unaided eye. The MIC was determined based on the guidelines of the Clinical Laboratory Standard Institute and The European Committee on Antimicrobial Susceptibility Testing EUCAST (Kahlmeter et al. 2006; Nour El Din et al. 2016). The micro-broth dilution method was implemented to determine MIC; Serial dilutions of AgNO_3_, OTC, AgNPs and Ag-OTC-Ns (100, 50, 25, 12.5 and 6.25 µg/ml) were used. The MIC was defined as the lowest concentration of Ag-OTC-Ns that completely inhibited visible growth of *K. pneumoniae* after incubation for 24 h at 37 °C. All MIC determinations were repeated in triplicate experiments independently.

### Determination of MBC

After the MIC determination of the AgNO_3_, OTC, AgNPs and Ag-OTC-Ns, aliquots of 10 µl samples from all the tubes which showed no visible bacterial growth, were seeded on Brain Heart Infusion Agar (BHI agar) plates and incubated for 24 h at 37 °C. When 99.9% of the bacterial population is killed at the lowest concentration of an antimicrobial agent, it is termed as the minimum bactericidal concentration (MBC) endpoint (Yusuf et al. 2022). This was done by observing pre and post-incubated agar plates for the presence or absence of bacterial colonies.

### Growth curve and time–kill study

Bacterial cultures were prepared to achieve a starting inoculum in exponential growth phase of 5 × 10^6^ CFU/ml (Zhao et al. 2020). Test tubes containing Mueller–Hinton broth with 1MIC concentration for AgNO_3_ (100 µg/ml), OTC (100 µg/ml), AgNPs (50 µg/ml) and Ag-OTC-Ns at both 1MIC (6.25 µg/ml) and 1MBC (12.5 µg/ml) were inoculated with overnight cultures of *K. pneumonia* clinical isolate. The cultures were then incubated in a shaker at 37 °C for 2, 4, 8 and 16 h. Samples were collected before adding tested samples (0 h) and after 2, 4, 8 and 16 h of incubation in the presence of AgNO_3_, OTC, AgNPs and Ag-OTC-Ns. Samples were serially (ten-fold) diluted before plating on agar and then viable counts, i.e. the number of CFU on the plates, were counted following overnight incubation at 37 °C (Mohamed et al. 2016). Experiments were performed using both a negative control (bacteria plus media) and a positive control (Ag-OTC-Ns plus media) (Reddy et al. 2014).

### In vivo antibacterial and histological assay

#### Animal ethical approve

Mice infection model was performed to investigate the attenuating potential of Oxytetracycline, Silver nanoparticles, and Silver Oxytetracycline Nano-structure on the pathogenicity of *K. pneumoniae*. All experimental protocols were done following the ethical standards adopted by National Center for Radiation Research and Technology Research Ethics Committee (REC-NCRRT-EAEA) (NO. 32A/23).

#### Mouse model of infection by *K. pneumonia*

After 1 week quarantined, 60 C57BL/6 male mice (25 ± 2.5 g) were randomly divided into six groups (n = 10): Sham group (administrated normal saline), Model group (infected without treatment), AgNO_3_ group (infected with AgNO_3_ treatment), OTC group (infected with OTC treatment), AgNPs groups (infected with AgNPs treatment) and Ag-OTC-Ns group (infected with Ag-OTC-Ns treatment). The infected groups were treated orally once day for 5 days with 0.1 ml of AgNO_3_, OTC, AgNPs and Ag-OTC-Ns according to group types (the dose given was depended on MIC and the weight of the mice body).

For induction of bacterial infection, mice were anesthetized by inhaling isoflurane, then the mice were held in the upright position with head up then 30 μL of 1 × 10^8^ CFU/ml *K. pneumoniae* clinical isolate suspension was dripped into the nasal cavity.

### Histopathological analysis

Lung tissue specimens were collected from all animal groups then fixed in 10% neutral buffered formalin. The fixed specimens were then trimmed, washed and dehydrated in ascending grades of alcohol, cleared in xylene, embedded in paraffin, sectioned at 46 μm thickness and stained by hematoxylin and eosin according to Salvi et al. (2021). Prepared slides sections were examined by light digital microscope (Olympus xc30. Tokyo. Japan). Each tissue section of lung was given a score from 0 to 4 based on the amount of area affected by interstitial inflammation, alveolar wall thickening, peribronchial inflammation and interstitial edema (0 ≤ 10%, 1 = up to 30%, 2 = up to 50%, 3 = up to 70%, 4 ≥ 70%) (Eldh et al. 2012).

### Determination of *K. pneumoniae* viable bacterial count

Lungs were removed aseptically and homogenized with a tissue homogenizer for up to 30 s in sterile glass tubes with 5 ml of sterile saline. The lung homogenate was vigorously agitated with a Vortex mixer to disrupt bacterial aggregates before the preparation was plated for CFU counting (Kostina et al. 2005). Homogenized lung samples were then serially diluted in sterile saline and plated on MacConkey agar plates and incubated for 18 to 20 h at 37 °C, and the numbers of viable bacterial CFU were determined. The data were expressed as log10 CFU per lung per mouse (means ± standard deviations).

### Measurement of the serum inflammatory biomarkers

Serum samples stored at  − 80 °C were used for the measurement of the levels of INF and IL-12 using fluorescent-labelled microspheres (FluorMAP System; R&D Systems, Wiesbaden-Nordenstadt, Germany) and the Luminex 100 instrument (Luminex BV, Oosterhout, the Netherlands). All procedures closely followed the manufacturer’s instructions.

### Acute toxicity of Ag-OTC-Ns

Acute Toxicity of Ag-OTC-Ns was estimated according to Ong et al. (2016) with some modification. Twenty adult Swiss albino mice weighting 20–25 g were maintained under standard laboratory conditions, such as temperature 20 °C ± 2 °C, humidity 45–55%, and a 12:12 h light/dark cycle. The animals had free access to standard pellet diet (Amrut feeds, Bangalore), with water provided ad labium under strict hygienic conditions. Animals were divided into five groups (n = 4) to estimate the acute toxicity at different concentrations upon oral administration as follows: Ag-OTC-Ns at a concentration of 6.25, 12.5, 25, 50, 100 µg/ml and control group, the control received tap water. The animals were observed for 14 days after drug administration to identify mortality, if any. The observations were made twice daily, one at 8 a.m. and another at 8 p.m.

### Statistical analysis

The differences in the means of the results between untreated and treated *K. pneumoniae* were analyzed by Student’s T-test. The probability value of p < 0.005 was considered significantly different.

## Results

### Ag-OTC-Ns preparation and validation

#### Ag-OTC-Ns preparation

**Fig. 1:**
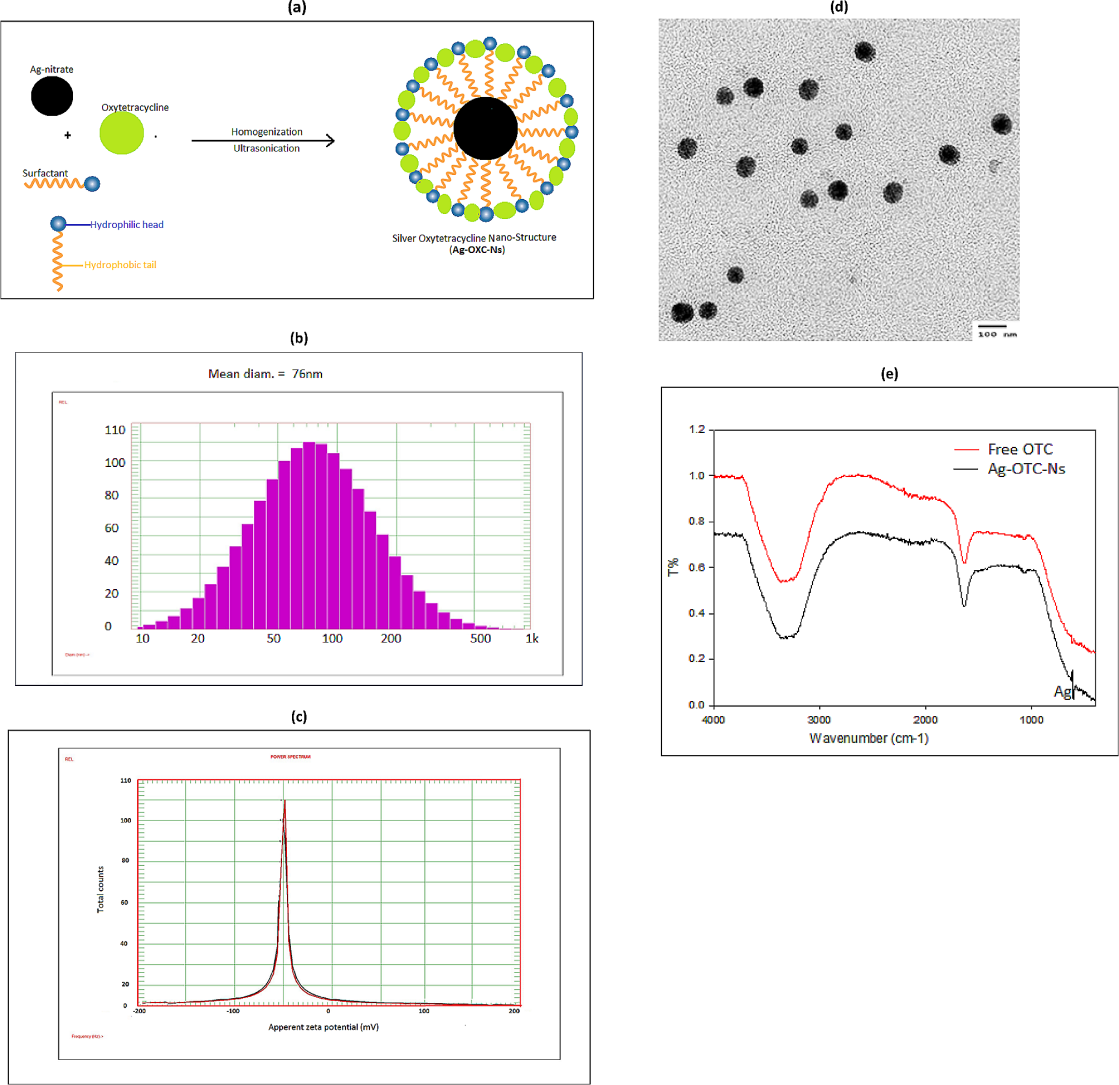
**a** Diagram of Ag-OTC-Ns preparation, **b **DLS diagram of Ag-OTC-Ns, **c** Zeta potential of Ag-OTC-Ns, **d** TEM of Ag-OTC-Ns, and **e** FT-IR of free OTC and Ag-OTC-Ns

Silver oxytetracycline Nano-structures (Ag-OTC-Ns) were prepared by simple homogenization-ultrasonication method in the presence of surfactant. Ag-OTC-Ns consists of silver nanoparticle as cores and oxytetracycline as outer Nano-shells Fig. 1a; Surfactants were used to create new kinds of improved highly ordered active Ag-OTC-Ns (Wei et al. 2019). It has been demonstrated that using Nano carriers (Nano-structure) for improves conventional antibiotics efficacy against XDR microorganisms (Ficai and Grumezescu 2017). The ability of these nanostructures to modify their geometry, shell morphology, and shell material had attracted a lot of attention because of their unique properties and potential applications ranging from antimicrobial and anti-inflammatory efficacy to drug delivery. Recent research suggests that nanostructured materials, including silver, gold, copper, bismuth, and other NPs, can enhance the antibacterial properties of traditional antibiotics when used in conjunction with Nano based drugs in low dosages.

### Ag-OTC-Ns validation

The size distribution and zeta potential analysis of Ag-OTC-Ns were performed using DLS Zeta Sizer Technique at neutral pH and the sample was diluted by deionized water. Dynamic Light Scattering (DLS) is a compelling estimation method utilized for measuring the hydrodynamic size of common nanomaterial (Salvi et al. 2021). Figure 1b shows Ag-OTC-Ns size distribution with 76 nm. Zeta potential is ordinarily measured to estimate the surface charge and the stability of nanomaterial’s (Honary and Zahir 2013). Figure 1c shows the zeta potential of Ag-OTC-Ns at about  −  50 mV. Particles with zeta potentials that are more negative than  30 mV are regularly considered stable (Larsson et al. 2012). High negative zeta potentials greater than  − 30 mV lead to monodispersity (uniform size in a dispersed phase) (Huo et al. 2019).

Figure 1d shows the TEM image of Ag-OTC-Ns that confirms the circle shape of particles with average size about 60 nm. Dynamic Light Scattering (DLS) estimates the hydrodynamic diameter of dissolved nanoparticles and TEM estimate particles size in solid state (Brar and Verma 2011). Size dispersed particle averages of DLS values are greater than TEM values; Because, DLS you measure hydration sphere diameter where there will be solvent molecules associated with your particle (Maguire et al. 2018).

Figure 1e shows FT-IR spectra of Ag-OTC-Ns this analysis was conducted to determine the molecular interaction between the AgNPs and OTC. The spectrum shows transmission one peak at 3372 cm^−1^ resulted from the stretching vibration of N–H bond; peaks at 627 cm^−1^ was due to the bending vibration of N–H bond, peak at 1634 cm^−1^ was attributed to the stretching vibrations of C=O bonds on the amide and the ring, and peak at 610 cm^−1^ indicated presence of silver nanoparticles. The peak ranged at range from 618 to 602 cm^−1^ indicates the vibration stretching of metal nanoparticle binding (Richardson et al. 1997).

### Gamma ray effect

Exposure of the Ag-OTC-Ns final product to different doses of gamma ray had resulted in no change in the stability up to 10 kGy. On the other hand, any further increase in doses had caused loss of nanostructure stability and finally precipitation of silver as dark precipitate. Therefore, use of large doses of gamma ray for sterilization of Ag-OTC-Ns should be avoided. Solution of Ag-OTC-Ns had showed no microbial growth after 5 kGy indicating a total sterile product.

### In vitro activity against *K. pneumoniae*

#### Measurement of inhibition zone diameter (IZD) and minimum inhibitory concentration (MIC)

The inhibition zone (IZ) and MIC values for AgNO_3_, OTC, AgNPs and Ag-OTC-Ns were determined according to CLSI. The IZ of AgNO_3_, OTC and AgNPs were 16 ± 0.12, 15 ± 0.021 and 18 ± 1.01 mm, respectively whereas Ag-OTC-Ns showed an IZ of 30 ± 0.014 mm against *K. pneumonia* isolate Table 1. The results indicated that Ag-OTC-Ns are more effective in compare with AgNO_3_, OTC and AgNPs against *K. pneumonia* isolate stain by at least 46.5, 50 and 40% respectively. The results of MIC in Table 1 shows that, Ag-OTC-Ns has MIC at 6.25 µg/ml, where AgNPs at 50 µg/ml and both AgNO_3_ and OTC at 100 µg/ml against *K. pneumonia* clinical isolate. This clearly illustrates the advantage of the combination between oxytetracycline and AgNPs as Ag-OTC-Ns over the other free experimental samples (AgNPs, AgNO_3_ and OTC). This significant antimicrobial activity of the Ag-OTC-Ns against *K. pneumoniae*, could be attributed to the interaction between the AgNPs and OTC that enhancing the penetration of antibiotics inside bacterial cell (Aabed and Mohammed 2021).

**Table 1:** Inhibition zone diameter (IZD), MIC and MBC

Stains	AgNO_3_	OTC	AgNPs	Ag-OTC-Ns
Inhibition zone (mm) at 100 µg/ml)				
*K. pneumonia ATCC 13883*	18 ± 0.32	17 ± 0.31	20 ± 1.02	31 ± 1.32
*K. pneumonia* clinical isolate	16 ± 0.12	15 ± 0. 21	18 ± 1.01	30 ± 0.014
MIC (µg/ml)				
*K. pneumonia ATCC 13883*	> 100	> 100	> 100	50
*K. pneumonia* isolate	100	100	50	6.25
MBC (µg/ml)				
*K. pneumonia ATCC 13883*	> 100	> 100	> 100	100
*K. pneumonia* clinical isolate	> 100	> 100	100	12.5

#### Measurement of MBC

After the MIC determination of the AgNO_3_, OTC, AgNPs and Ag-OTC-Ns, aliquots of 10 µl samples from all the tubes which showed no visible bacterial growth, were seeded on Brain Heart Infusion Agar (BHI agar) plates and incubated for 24 h at 37 °C.

After 24 h of incubation at 37 °C, the suspension from the tubes of Ag-OTC-Ns at 12.5, 25 and 50, 100 µg/ml, was observed no growth of bacteria in all the concentrations hence confirming its bactericidal activity. AgNPs displayed MBC at 100 µg/ml but AgNO_3_ and OTC had MBC at concentration more than 100 µg/ml. Whereas Ag-OTC-Ns has MBC at concentration 12.5 µg/ml (Table 1) against *K. pneumonia* clinical isolate, presenting an 87.5% decrease lower than other AgNPs, AgNO_3_ and OTC. Nanoparticles provide a greater and better bactericidal effect due to their larger surface area (Doty et al. 2005).

### Time kill experiments with *K. pneumoniae*

**Fig. 2:**
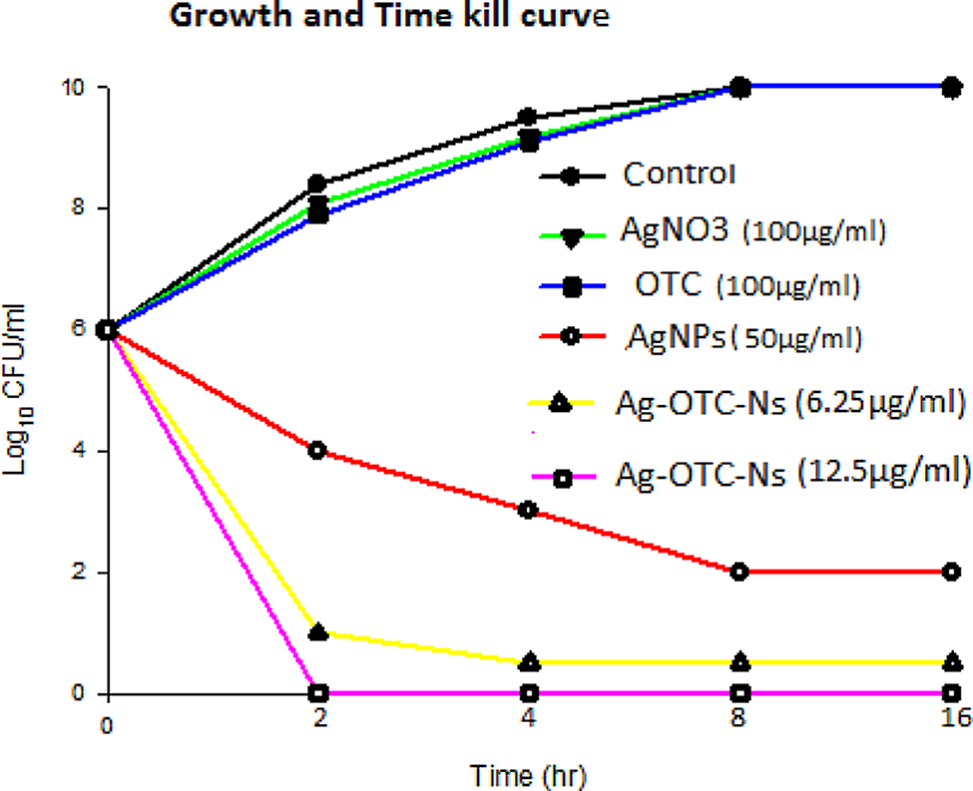
Growth and time killing curve of *K. Pneumoniae* clinical isolate

The time kill curve of *K. pneumoniae* clinical isolate stain for selected regimens of AgNO_3_, OTC, AgNPs and Ag-OTC-Ns are shown in Fig. 2. When used alone, AgNO_3_ and OTC at 100 µg/ml had no effect on bacterial growth. The initial reduction of bacterial growth was observed with AgNPs after 2 h and endpoint of inhibits bacterial growth is after 8 h at 50 µg/ml concentration. Bacterial growth inhibition with Ag-OTC-Ns was very rapid at 1MIC (6.25 µg/ml) after 2h and endpoint of bacterial growth inhibition is after 4 h. Despite complete initial killing of bacterial cell, is observed with Ag-OTC-Ns at 2MBC (12.5 µg/ml) after 2 h. Time-kill studies showed that endpoint of bacterial growth inhibition for AgNPs achieved after 8 h at 50 µg/ml, while Ag-OTC-Ns appear after 4 h at 6.25 µg/ml and after 2 h at 12.5 µg/ml respectively, whoever; AgNO_3_ and OTC has no effect on *K. Pneumoniae* growth. Therefore, these results show that Ag-OTC-Ns inhibit growth and kill the bacterial cell of *K. pneumonia* clinical isolate at low concentration and faster than AgNPs, AgNO_3_ and OTC.

### In vivo activity against *K. pneumonia*

#### Histopathological analysis of pulmonary lesions

**Fig. 3:**
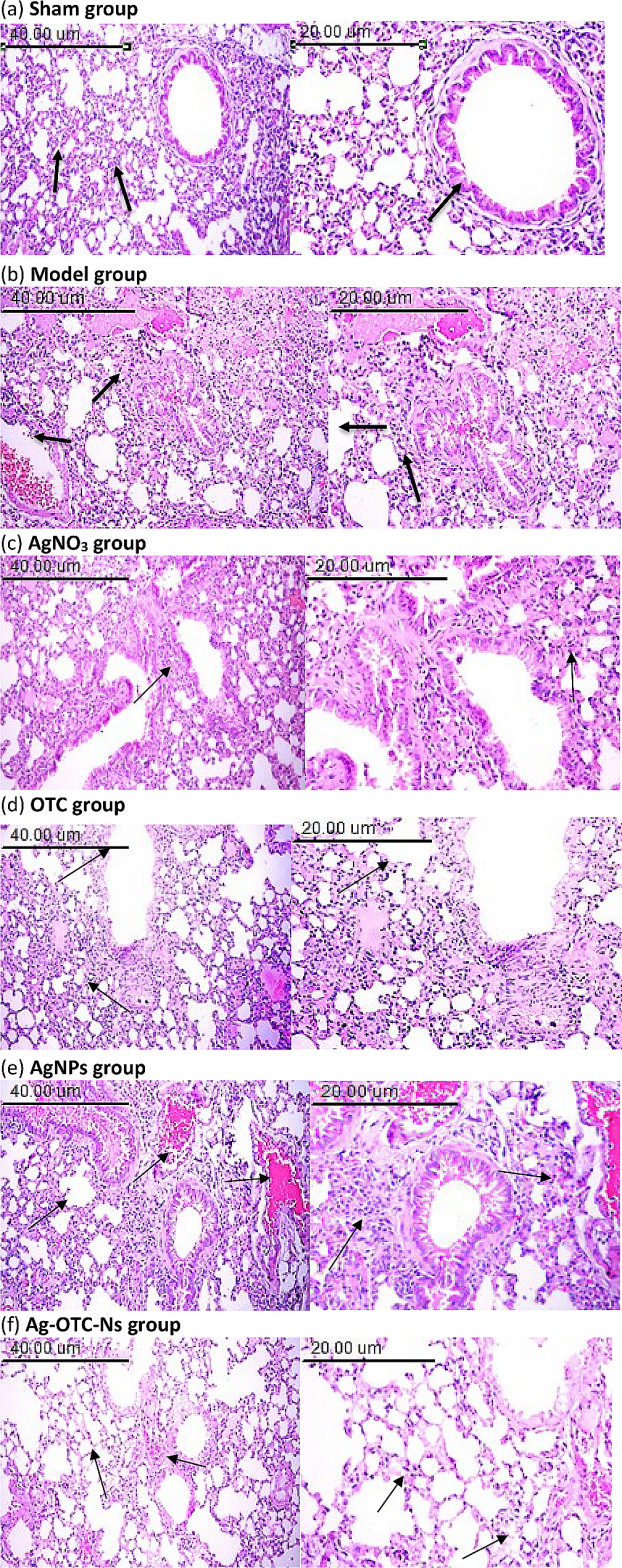
**a** is Model group; showing normal archicture of alveoli and interstitium arrow (× 100) and normal hjstologicla structure of bronchiolar epithelial lining arrow (H&E × 200), **b** is Sham group; showing photomicrograph of lung tissue section showing: thickening of alveolar wall with congestion of peribronchial arteriols arrow (× 100) and focal emphysematous area with mononuclear cells infiltration of interstitium arrow (H&E × 200), **c** is AgNO_3_ group; showing photomicrograph of lung tissue section showing: cellular infiltration of peribronchial and perivascular spaces arrow (× 100) and severe thickening of alveolar wall arrow (× 100) (H&E × 200)**, d** is OTC group; showing photomicrograph of lung tissue section showing: huge numbers of cellular infiltration of peribronchial and perivascular spaces arrow and multiple focal emphysematous areas accompanied with moderate thickening of alveolar wall arrow (× 100) (H&E × 200), **e** is AgNPs group; showing photomicrograph of lung tissue section showing: thickening of alveolar wall with congestion of peribronchial arteriols arrow (× 100) and mononuclear cells infiltration of interstitium and peribronchial spaces arrow (H&E × 200), and **f** is Ag-OTC-Ns group; showing Photomicrograph of lung tissue section showing: normal archicture of alveoli with congestion of peribronchial arterioles arrow (× 100) and few numbers of mononuclear cells infiltrate alveolar wall arrow (H&E × 200)

Examination of the lung tissues of sham group showed normal histological structure of lung lobules. Airspaces and alveoli were separated by fine delicate inter-alveolar septa, and normal vasculature with scant perivascular connective tissue was seen. The alveoli appeared inflated with thin inter-alveolar septa beside to intact bronchiolar epithelial lining Fig. 3a. Lung tissue section of animals exposed to infection without treatment (Model group) revealed focal necrotic areas with diffuse cellular infiltration of the interstitium by mononuclear cells mainly lymphocytes and macrophages in peribronchial and perivascular spaces with congestion of blood capillaries. Marked thickening of the inter-alveolar septa and with complete loss of bronchial goblet cells were detected. Multiple focal emphysematous areas accompanied with giant alveoli formation were notice Fig. 3b.

Animal’s group infection followed by treatment with AgNO_3_ (AgNO_3_ group) revealed nearly the similar picture of untreated group. Massive inflammatory cells infiltration mainly lymphocytes and macrophages in alveolar wall and perivascular spaces, Marked congestion of peribronchial arterioles were noticed Fig. 3c. On the other hand, animals' group of infection followed by treatment with free oxytetracycline demonstrated the histological finding of severe cellular infiltration of the interstitium by mononuclear cells in peribronchial and perivascular spaces as well as thickening of the inter-alveolar septa with multiple focal emphysematous areas Fig. 3d. Lung tissue section of animals group infection followed by Ag nanoparticles treatment showed moderate numbers of lymphocytes and macrophages infiltrate the interstitium, peribronchial and perivascular spaces were seen. Moderate thickening of the inter-alveolar septa and complete loss of bronchial goblet cells were detected. Multiple focal emphysematous areas accompanied with congestion of peribronchial arterioles were notice Fig. 3e. Lung tissue section of animals group infection followed by Ag-OTC-Ns treatment showed few numbers of inflammatory cells in peribronchial spaces. Minimum thickening of the inter-alveolar septa; Focal emphysematous areas accompanied with giant alveoli formation were seen Fig. 3f.

### Scoring of pathological lesions

Following *K. pneumoniae* infection, pathological injuries, such as lung tissue structure destruction, alveolar wall thickening and fusion, pulmonary interstitial edema, hemorrhage, and inflammatory cell infiltration, were observed, and Histopathological scores of lung tissue sections were evaluated and recorded with scores 0 to 4 (Table 1 Supplementary data). The histopathological score was related to the following distribution: thickened alveolar walls; edema; and tissue parenchymatous lesions such as congestion and hemorrhage. Tissue sections were examined with the following scores: 0, no pathological lesion; 1, minimum; 2, mild; 3, moderate; and 4, severe. In this study, we screened that AgNO_3_, OTC, AgNPs and Ag-OTC-Ns targeted *K. pneumoniae* isolates and demonstrated that Ag-OTC-Ns therapy, effectively rescued mice with prevalent *K. pneumoniae* isolate caused pneumonia in the early stage and has the score 1, in compere with AgNO_3_, OTC, AgNPs.

### *K. pneumoniae* pulmonary cell count

Colony forming units (CFUs) of pulmonary *K. pneumoniae* tested strain were significantly reduced when exposed to Ag-OTC-Ns treatments; in contrast to other groups treated with AgNO_3_, OTC, and AgNPs. This resulted from a significant synergistic interaction between Ag nanoparticles and Oxytetracycline (Table 2 Supplementary data).

### Inflammatory biomarkers analysis

The *K. pneumoniae* pulmonary infections are implied by inflammation caused by lipopolysaccharide (LPS), the major component of Gram negative bacteria outer membrane and the main cause of the pathophysiological progressions of septicemia, toxic shock syndrome (TSS), and inflammation (Martin and Bachman 2018; Ramos-Castañeda et al. 2018). Therefore, the recent advanced treatment regimens for *K. pneumoniae* should include antibacterial as well as anti-inflammatory (Özçelik et al. 2008). In the recent study, Ag-OTC-Ns could effectually diminish the levels of Interferon Gamma and IL-12, and as a result it could effectively lower lung damage in *K. pneumoniae* infected mice Table 2. In model group, mice died prior to the last day of the experiment. Inflammatory biomarkers assessment for Interferon Gamma and IL-12 revealed their lowering to approximately normal level. Due to their anti-inflammatory, antiviral, and antibacterial properties, of Nano silver solutions may be useful in treating respiratory infections and inflammation brought on by viruses and/or microorganisms (Nadworny et al. 2023).

**Table 2:** Hematological analysis (serum biomarkers)

Group	Sham	Model	AgNO_3_	OTC	AgNPs	Ag-OTC-Ns
Interferon Gamma Pg/ml	51.3	121	127.3	138	75.4	52.8
IL-12 Pg/ml	38.2	92	86.5	89.5	61.1	40.5

### Toxicity of Ag-OTC-Ns

Using of Nano scaled materials are quickly expanding, but the only limitation is their conceivable ecotoxicological impacts which are still not absolutely known. The results confirmed that no dead mice were observed along the experimental period, there was no sign of behavioral abnormality in the surviving mice indicating that the Ag-OTC-Ns are safe at the concentration used. The results illustrate that Ag-OTC-Ns displayed no toxicity in mice; our investigations unequivocally appear that Ag-OTC-Ns can be safely utilized for wide range as antimicrobial agents for both in vitro and in vivo applications.

## Discussion

Recent research suggests that nanostructured materials, including silver, gold, copper, bismuth, and other NPs, can enhance the antibacterial properties of traditional antibiotics when used in conjunction with Nano based drugs in low dosages (Hochvaldová et al. 2022). It has been demonstrated that using Nano-carriers (as Nano-structure) for conventional antibiotics improves their antibacterial efficacy against drug resistant microorganisms (Ficai and Grumezescu 2017). Adhesion of nanostructures to microbial cell surfaces, causes numerous cell wall deformation at sites of adhesion (Luan et al. 2018). Being Nano-structured drugs can enter within the tissue system, facilitate simple take up of the drug by cells, and allow an efficient drug delivery at target sites (Patra et al. 2018).

Dynamic Light Scattering (DLS) estimates the hydrodynamic diameter of dissolved nanoparticles and TEM estimate particles size in solid state (Brar and Verma 2011). The size of dispersed particle averages of DLS values is greater than TEM values; Because, DLS you measure hydration sphere diameter where there will be solvent molecules associated with your particle (Maguire et al. 2018) and DLS measures the hydrodynamic size of suspended particles within a liquid, and therefore the size of the particles is larger compared to measuring the same particles using TEM (El-Batal et al. 2021; Lim et al. 2013).

High negative zeta potentials lead to monodispersity (uniform size in a dispersed phase) (Huo et al. 2019). The zeta potential is an imperative factor for assessing the steadiness of a Nano-form; It may be a work of the molecule surface charge, which balances the greatness of the electro-static repugnance between particles (Mukhopadhyay 2022). TEM reveals the structure of molecule from interior and gives thought approximately molecule diameter and framework structure (Salvi and Pawar 2019). The peak ranged from 618 to 602 cm^−1^ indicates the vibration stretching of metal nanoparticle binding (Richardson et al. 1997). The bind via the electrostatic attraction and repulsion forces between metallic nanoparticles and antibiotic are responsible for stabilization of the Ns and prevents Ag-OTC-Ns separation and precipitation.

Gamma radiation more than 10kGy, causes degradation and separation of Ns system, because high doses of gamma ray cause excess generation of oxygen, hydrogen, and peroxide radicals that enhance random movements of particles in solution, leads to aggregation and agglomeration of NPs (Mosallam et al. 2021) and finally precipitation of silver as dark precipitate.

The in vitro activity of Ag-OTC-Ns against *K. pneumonia* demonstrates a greater efficacy than free AgNO_3_, OTC, and AgNPs. Because, the combination of antibiotics and metals NPs has many advantage; can return antibiotics activity, cause reduction in therapeutic doses and toxicity of antibiotics, decrease development of antibiotics resistance and treatment duration time (Shabatina et al. 2023).This improvement in activity may be attributed to the interaction between the NPs and antibiotic (Lopez-Carrizales et al. 2018), that enhancing the penetration of antibiotic inside bacterial cell (Aabed and Mohammed 2021) and promotes an increase in the antibiotics local concentration at the site of action in bacterial cell (Allahverdiyev et al. 2011). Formation of Ns, can synergist antimicrobial efficacy of antibiotics against *K. pneumonia*, where the penetration of antibiotics is more possible by metal antibiotic nanostructure formation (Aabed and Mohammed 2021).

The previous studies refer to synergistic effect of AgNPs-antibiotic conjugation, to reduce the bacterial cell count at lower antibiotic concentration (Hwang et al. 2012). A recent study confirmed the synergistic combination of antibiotics and AgNPs against *E. coli* and *Salmonella spp.* (Abo-Shama et al. 2020). The formation of novel nanostructures provide disrupted antibacterial effects, including better biocompatibility, in contrast to free antimicrobial agents, which have poisonous and short lived functions (Modi et al. 2022).The silver ions produced from AgNPs also cause significant cytotoxicity of eukaryotes, Thus, to increase the biosafety in vivo, most research overcome this problem via AgNPs conjugation (Yang et al. 2021). Nanoparticles can act by delivering antibiotic and possessing intrinsic antimicrobial activity (Sayed et al. 2022). Other study confirm inhibition of Carbapenem resistant strain of *Acinetobacter baumannii* infection in human pulmonary epithelial cell by polyvinylpyrrolidone Capped AgNPs (Tiwari et al. 2017). The use of Ag-OTC-Ns formulation is a promising method to increase AgNPs and OTC penetration because the surfactant included in the preparation that have hydrophobic and hydrophilic nature (Ferreira et al. 2021).

According to results of this study, Ag-OTC-Ns have MIC at lower concentration than occur with AgNO_3_, AgNPs and OTC, which reinforces the idea of Nanostructure formation. Due to the metals NPs have various targets site on bacterial cell, his makes the bacterial resistance to it very weak than antibiotics (Aabed and Mohammed 2021). In a prior study, combination of AgNPs with methicillin resulted in a lowering of the MIC against *Staphylococcus epidermidis* from 250 to 7.8 µg/ml (Thomas et al. 2020). Previous studies confirm free oxytetrcycline had a minimum inhibitory concentration of 150 µg/ml, while metal conjugated oxytetrcycline functioned better at a very low inhibitory concentration of 7.02 µg/ml. (Sarkar et al. 2022).

The MIC was decreased by the antibiotic nanoparticles combination; this value was more significant for nanoparticles carrying both ciprofloxacin and imipenem against *Staphylococcus aureus* (Shafiei et al. 2020). The combination of AgNPs with Imipenem caused MIC assays of antibiotic concentrations to decrease roughly by a range of 16 to 256 fold (Fontoura et al. 2023). The presence of surfactant vesicles might lower the MIC values of antibiotics more than free (Abo Kamer et al. 2023). Nano-systems for antibiotic delivery and targeting to infection sites offer some advantages, such as improved solubility, increased stability, improved epithelium permeability and bioavailability, prolonged antibiotic half-life, tissue targeting, and minimal side effects (Yeh et al. 2020). Nanostructures can deliver antimicrobial agents to target the infected sites and reduce the dosage and toxicity of antibiotics (Lee et al. 2019).

In this study, the investigated killing activity of Ag-OTC-Ns against *K. Pneumoniae*, demonstrated Ag-OTC-Ns can be kill the *K. Pneumoniae* isolates in a shorter time at low concentrations in compere with AgNO_3_, AgNPs, and OTC. Due to their potent biocidal impact against microorganisms, silver nanoparticles are widely recognized as the most effective antimicrobial agents (Oei et al. 2012). Meropenem conjugated nanomaterial’s can successfully kill *K. pneumoniae* that is regarded as Meropenem resistant (Galbadage et al. 2019). Nanoparticles has smaller size with large surface area so are more toxic to bacteria and exhibit superior bactericidal effects (Zhang et al. 2016). NPs’ precise antibacterial mechanisms are still unknown. However, other studies have hypothesized that the ability of NPs to enter cells, creation of free radicals, inactivation of proteins in cells, development of reactive oxygen species (ROS) and finally kill the bacterial cell (Dakal et al. 2016). The combination of Metals NPs with commercial antimicrobial drugs (e.g., antibiotics, antifungals, and antivirals) may offer several opportunities to overcome some disadvantages of their individual use and enhance effectiveness (Ribeiro et al. 2022).

Antibiotic resistance has increased as a result of the misuse of antibiotics in both human and animal disease management; extensively drug resistant (XDR) cases of *K. pneumoniae* have been documented frequently (Abushaheen et al. 2020; Khairy et al. 2020). Due to their distinctive physiochemical characteristics, nanoparticles can circumvent antibiotic resistance mechanisms and use a variety of novel bactericidal pathways to produce antimicrobial action (Gupta et al. 2019). Lipopolysaccharide (LPS), the major component of gram negative bacteria outer membrane and the main cause of the pathophysiological progressions of septicemia, toxic shock syndrome (TSS), and inflammation, is implicated in the *K. pneumoniae* lung infections (Martin and Bachman 2018).

The results demonstrated that Ag-OTC-Ns is effective and can prevent pneumonia in infected mice by *K. pneumoniae* isolate in the early stage and has the minimum lung tissue structure destruction, alveolar wall thickening and fusion, pulmonary interstitial edema, hemorrhage, and inflammatory cell infiltration (score 1), in compere with AgNO_3_, OTC, and AgNPs. Nanotechnology will provide a viable alternative method for the development of a long term strategy to tackle the problems of diagnosis and drug delivery in pulmonary infections (Ingle et al. 2016). By combining several nanostructures, traditional respiratory therapy medications can be delivered, transported, and bioavailability become better than when they were used alone (Ibarra-Sánchez et al. 2022).

AgNPs also exhibited anti-inflammatory effects by suppressing the production of pro-inflammatory cytokines (IL-1β, IL-6 and TNF-α) in macrophages (Tyavambiza et al. 2021). NPs also have direct anti-inflammatory effects via regulating inflammatory mediators and immune cells (Huang et al. 2024). The AgNPs also demonstrated anti-inflammatory effect by preventing the release of cytokines that promote inflammation (TNF-alpha, IL-6, and IL-1 beta) in addition to antibacterial activities (Tyavambiza et al. 2021). Due to their anti-inflammatory, antiviral, and antibacterial properties, of Nano silver solutions may be useful in treating respiratory infections and inflammation brought on by viruses and/or microorganisms (Nadworny et al. 2023). Metal and metal oxide nanoparticles dominate nanostructures that are designed to improve them antimicrobial and anti-inflammatory effects (Granados et al. 2021). Suggested that the enhanced apoptosis of inflammatory cells and decreased levels of pro-inflammatory cytokines were responsible for Nano silver anti-inflammatory capabilities (Shin et al. 2018). Respiratory tract infections are treated using a variety of nanotechnology applications, from early diagnosis to treatment plans (Chen et al. 2022).

The new prepared Ag-OTC-Ns has low or no significant toxicity as mentioned in results because; Nanostructure or Nano-coating formation is approaches that reduce the toxicity of metal based NPs due to surface chemistry alterations of metal NPs (Zhang et al. 2022). Using suitable surface changes is an alternative strategy to control the characteristics of metallic nanoparticles, including reducing their toxicity (George et al. 2012). Metallic nanoparticles can have their surfaces modified to create totally new and less toxic metal drug Nano-combination (Długosz et al. 2020). Nanostructure reduces the risk of NPs used in industry and biology and provides the potential to create novel and effective Nano-drugs (Awashra and Młynarz 2023). The toxicity of metal nanoparticles can be inhibited by combining them with other substance, this decrease the NPs ability to interact with living things, and accumulate in tissues and organs (Sukhanova et al. 2018).

Nanostructures serves as a physical boundary that ought to minimize water interaction with the nanomaterial surface and the discharge of poisonous particles from the center fabric to connected with life forms (Buchman et al. 2019). Antibiotics can be combined with NPs as nanostructure form to overcome toxicity of antibiotics and metals (Adeniji et al. 2022). Nano antibiotics have been reported to be more efficient, durable and less toxic (Modi et al. 2022). Drug dosage and toxicity can be reduced using nanoparticles (Kotrange et al. 2021).

This present work emphasized the preparation, validation, and antibacterial properties of Ag-OTC-Ns. Synthesized Ag-OTC-Ns were characterized by DLS, TEM and FT-IR. Furthermore, the Ag-OTC-Ns were assessed for antimicrobial activity against *K. pneumonia* stander and clinical isolates. Ag-OTC-Ns exhibited excellent antibacterial activity at 6.25 μg/ml. Ag-OTC-Ns exhibited bactericidal effect at 12.5 μg/ml and significant eradication of *K. pneumonia* in compere with free OTC, AgNPs and AgNO_3_. Ag-OTC-Ns displayed no toxicity in mice, therefore, the Ag-OTC-Ns might be useful for various fields like pharmaceutical products, drug delivery, and many other commercial processes.

### Supplementary Information

Additional file1 (DOCX 24 kb)

## Data Availability

The data and materials that support the findings of this study are available from the corresponding author, upon reasonable request.

## Declarations

### Constant of participate and publish

All authors agree to participate and publish.
