# Supplementary material for: Eradication of Klebsiella pneumoniae pulmonary infection by silver oxytetracycline nano-structure
Source: AMB Express. 2024 May 29;14:62. doi: 10.1186/s13568-024-01720-5 (PMC11136936; doi:10.1186/s13568-024-01720-5)
Supplement: Supplementary file 1 — Additional file1 (DOCX 24 kb) [file 13568_2024_1720_MOESM1_ESM.docx]

**Table (1 Supplementary data): Scoring of pathological lesions**

| Group | Sham | Model | AgNO_3_ | OTC | AgNPs | Ag-OTC-Ns |
| --- | --- | --- | --- | --- | --- | --- |
| Score | zero | 4 | 4 | 4 | 3 | 1 |

**Where;** 0, no pathological lesion; 1, minimum; 2, mild; 3, moderate; and 4, severe lung lesions

**Table (2 Supplementary data): *K. pneumoniae* pulmonary cell count load (CFU/g Lung) (mean SD)**

| Group | Sham | Model | AgNO_3_ | OTC | AgNPs | Ag-OTC-Ns |
| --- | --- | --- | --- | --- | --- | --- |
| Score | <100 | Death | > 9 x 10^7^ | > 4x 10^8^ | 6 x 10^6^ | 2x10^2^ |
